# Safety and immunogenicity of an MF59™-adjuvanted subunit influenza vaccine in elderly Chinese subjects

**DOI:** 10.1186/1742-4933-5-2

**Published:** 2008-02-20

**Authors:** Rongcheng Li, Hanhua Fang, Yanping Li, Youping Liu, Michele Pellegrini, Audino Podda

**Affiliations:** 1 Centre for Vaccine Clinical Research, Centers for Disease Prevention and Control of Guangxi Zhuang Autonomous Region, 18 Jinzhou Street, 530022 Nanning City, China; 2 National Institute for the Control of Pharmaceutical and Biological Products (NICPBP), Temple of Heaven, Beijing, China; 3 Centers for Disease Prevention and Control of Wuzhou, Guangxi Zhuang Autonomous Region, China; 4 Clinical Research Development and Medical Affairs, Novartis Vaccines and Diagnostics, Siena, Italy

## Abstract

**Background:**

The safety and immunogenicity of an MF59™-adjuvanted subunit influenza vaccine (Sub/MF59™; FLUAD^®^, Novartis Vaccines) was evaluated among elderly Chinese subjects (≥ 60 years of age). After a preliminary Phase I, open-label study (n = 25) to assess safety 1–14 days post-vaccination, a comparative observer-blind, randomised, controlled clinical trial (n = 600) was performed to assess safety and immunogenicity versus a non-adjuvanted subunit influenza vaccine (Subunit; Agrippal^®^, Novartis Vaccines). Subjects were randomised (2:1) to receive Sub/MF59™ or Subunit.

**Results:**

Both vaccines were well tolerated, with no vaccine-related serious adverse events reported during the Phase I trial. During the observer-blind study, local and systemic reactions were generally similar for both vaccines 1–22 days post-vaccination; however, injection-site induration was more frequent among the Subunit group (P < 0.05), and mild pain at the injection site and fever were more frequent among Sub/MF59™ recipients (P ≤ 0.005). Both vaccines induced a significant (P < 0.001) increase in geometric mean titres (GMTs) for the three strains tested, versus baseline; GMTs against A/H1N1, A/H3N2 and B were significantly higher in the Sub/MF59™ group (P = 0.034, P < 0.001 and P = 0.005, respectively). GMT ratios against A/H1N1, A/H3N2 and B were also significantly higher in the Sub/MF59™ group (P = 0.038, P < 0.001 and P = 0.006, respectively). Similarly, the percentage of subjects achieving seroprotection or seroconversion on Day 22 was greater for Sub/MF59™ recipients, reaching significance for A/H3N2 (P < 0.001).

**Conclusion:**

MF59™-adjuvanted subunit influenza vaccine is well tolerated by elderly Chinese subjects and induces a higher level of immunogenicity than a non-adjuvanted subunit influenza vaccine in this population that is at high risk of influenza-related complications.

**Clinical trial registry:**

, NCT00310648

## Background

Influenza infection represents a considerable global burden, affecting 5–15% of the adult population during annual influenza epidemics [1]. Each year, 3–5 million cases of severe illness and 250,000–500,000 deaths are thought to result from these epidemics worldwide [1,2].

South-east Asia is considered to be the global influenza epicentre, with several pandemic and epidemic strains known to have originated from China and Hong Kong since 1957 [3-5]. Furthermore, China is generally acknowledged as an area with a high influenza attack rate, due to the high population density and year-round circulation of the virus in tropical regions [6,7]. In China, pneumonia and influenza combined ranks fourth in the leading causes of death in adults (≥ 40 years of age) [8].

Due to their weakened immune response, the elderly are at increased risk of influenza and its related complications [9]. In China, the annual mortality rate for pneumonia and influenza has been reported to rise to 227.4/100,000 persons in the elderly (≥ 65 years of age), representing a 5-fold increase from the age-standardised annual mortality rate of 43.9/100,000 persons [8]. Furthermore, during 1999, the reported number of influenza-associated deaths in Hong Kong from cardiorespiratory disease, pneumonia and influenza, chronic obstructive pulmonary disease and ischaemic heart disease was higher in the elderly (1697 deaths), compared with adults (205 deaths; 40–64 years of age) [10]. Peaks in influenza circulation have been shown to coincide with increased mortality from these conditions, with the elderly accounting for approximately 70–90% of the associated deaths [10]. In addition to an increased number of deaths [10,11], the hospitalisation rate for pneumonia and influenza is also high in the elderly [12].

Influenza vaccine effectiveness has been studied extensively, mainly in temperate regions. Conventional influenza vaccines confer protection against laboratory-confirmed influenza in 70–90% of young adults (18–64 years of age) [13,14]; however, these vaccines are less effective (17–53%) in the elderly, due in part to the elderly's waning immunity [15]. When evaluating vaccine response, however, it is important to also consider racial background. For example, among a racially diverse, healthy elderly population in the USA, a reduced response to influenza vaccine was observed among elderly African Americans, compared with elderly Caucasian and Latinos [16]. Furthermore, racial differences in relation to vaccination have been observed in Taiwan among Han Chinese children compared with Aboriginal children; higher titres against hepatitis B vaccination were reported for Han Chinese children [17].

In Hong Kong, Taiwan and China, vaccination has been shown to offer protection against influenza [18-20]. Influenza vaccination prevented approximately 69% of influenza-related hospitalisation admissions in Hong Kong during the 2003–2004 winter season [19] and was strongly associated with a reduction in pneumonia, heart disease, stroke, diabetes mellitus and renal disease in Taiwan in 2001 [20]. Vaccine effectiveness, however, has also been reported to be lower in elderly Chinese people than in adult Chinese people (68.6% versus 74%, respectively) [18]. Despite the availability of an effective vaccine, coverage rates are low in China; for example, the general vaccination rate among the urban population of Beijing is only 10.5%, falling to 7.9% for those ≥ 60 years of age [21]. Thus, because currently available vaccines do not offer optimal protection in the elderly [14,15] and vaccine coverage rates are low [21], elderly Chinese people are at increased risk of influenza and its related complications. As China has the world's largest population of elderly people (>80 million) [22], influenza represents a considerable health and economic burden.

To meet the global challenge presented by waning immunity in the elderly, vaccines that offer the elderly enhanced immunogenicity and increased clinical protection are required. Addition of the adjuvant MF59™ [23] to subunit influenza vaccine (MF59™-adjuvanted subunit influenza vaccine; FLUAD^®^, Novartis Vaccines) has been shown to enhance the immune response and offer increased clinical protection in elderly subjects, compared with non-adjuvanted subunit influenza vaccine [24-26]. Furthermore, enhanced immunogenicity is observed in elderly subjects with underlying chronic conditions, who are at especially high risk of influenza and its complications [27]. Vaccination of elderly subjects with the MF59™-adjuvanted subunit influenza vaccine also confers protection against a broader range of influenza virus strains than non-adjuvanted subunit influenza vaccine [28,29] and has been associated with a reduced risk of hospitalisation for pneumonia and cerebrovascular disease in non-institutionalised elderly subjects [30,31].

In China, the registration of FLUAD^® ^for use in elderly people is currently under consideration. For this reason, a randomised comparative trial was performed in a large cohort of elderly Chinese people to assess the safety and immunogenicity of FLUAD^® ^against a non-adjuvanted subunit influenza vaccine.

## Results

**Figure 1:**
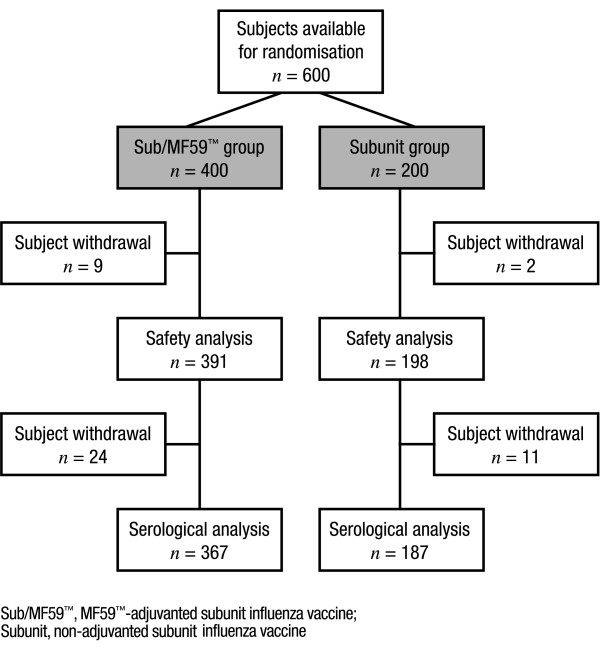
Subject participation in the Phase II/III trial.

All subjects (n = 25) enrolled in the Phase I open-label trial completed the safety evaluation. One subject was excluded from the analysis due to administration of Subunit vaccine, rather than Sub/MF59™. For the Phase II/III trial, the baseline characteristics of the study population were similar for the Sub/MF59™ and Subunit vaccine groups, with an equal match for gender, age and availability of current and past medical history. A total of 600 subjects were recruited in the subsequent Phase II/III trial and randomised to receive Sub/MF59™ (n = 400) or Subunit (n = 200) vaccine. Safety evaluation was completed for 589 subjects and serological analysis was performed for 554 subjects (Figure 1). The major reasons for patient withdrawal were refusal to continue the study (25 and 10 subjects receiving Sub/MF59™ and Subunit, respectively), a change in residence or hospital/being unable to receive follow-up as too busy (4 patients, 2 in each vaccine group) or other drop-out (6 and 1 subjects receiving Sub/MF59™ and Subunit, respectively). No subjects were withdrawn from the study because of an AE due to the study vaccines.

### Safety

Both vaccines were well tolerated by elderly subjects during the clinical trials. No vaccine-related serious adverse events (SAEs) were reported for Sub/MF59™ during the Phase I trial, which was a pre-requisite for continuation with the Phase II/III trial.

In the Phase II/III trial, the overall incidence of systemic reactions and AEs was generally similar between the Sub/MF59™ and Subunit groups; however, the incidence of local reactions was significantly (P = 0.012) higher in the Sub/MF59™ group compared with the Subunit group (24.0% versus 15.2%, respectively) (Table 1). Induration at the injection site was significantly (P < 0.05) more frequent in the Subunit group compared with the Sub/MF59™ group (2.5% versus 0.5%, respectively) (Table 2). Pain at the injection site and fever (axillary temperature >38°C) were significantly (P ≤ 0.005) more frequent in the Sub/MF59™ group compared with the Subunit group (10.2% versus 3.0% and 15.9% versus 7.6%, respectively) (Table 2). Local and systemic reactions were generally mild or moderate and transient. In particular, fever was mainly classed as mild or moderate in the Sub/MF59™ and Subunit groups and pain was classified as mild. No vaccine-related SAEs were reported during this large study for the Subunit group; however, a vaccine-related SAE (high fever) was reported for one subject (0.3%) in the Sub/MF59™ group (Table 1).

**Table 1:** Incidence of adverse events (AEs), adverse reactions and serious AEs (SAEs) for the two vaccine groups

	**Incidence, %(n)**		**P-value**
		
	**Sub/MF59™ (n = 391)**	**Subunit (n = 198)**	
**Local reactions**	24.0(94)	15.2(30)	0.012
**Systemic reactions**	10.7(42)	9.6(19)	0.666
**AEs**	6.1(24)	6.6(13)	0.840
**SAEs**	1.0(4)	0.5(1)	0.668
**Vaccine-related AEs (definitely, probably or possibly related to the vaccine)**	3.8(15)	5.1(10)	0.490
**Vaccine-related SAEs (definitely, probably or possibly related to the vaccine)**	0.3(1)	0.0(0)	1.000

Sub/MF59™, MF59™-adjuvanted subunit influenza vaccine; Subunit, non-adjuvanted subunit influenza vaccine

**Table 2:** Incidence of local and systemic reactions reported for the two vaccine groups

		**Sub/MF59™ %(n)**	**Subunit %(n)**
Local reaction	*Rash*	0.0(0)	0.5(1)
	*Erythema*	1.5(6)	1.5(3)
	*Induration*	0.5(2)	2.5(5)*
	*Swelling*	2.8(11)	1.0(2)
	*Pain*	10.2(40)**	3.0(6)
	*Pruritus*	1.3(5)	3.0(6)
Systemic reaction	*Headache*	3.6(14)	2.5(5)
	*Fever*	15.9(62)**	7.6(15)
	*Tiredness*	3.3(13)	1.0(2)
	*Diarrhoea*	0.8(3)	2.0(4)
	*Vomiting*	1.0(4)	1.0(2)
	*Myalgia*	1.8(7)	0.5(1)
	*Cough*	2.0(8)	2.0(4)
	*Angina*	1.5(6)	1.0(2)
	*Watery nasal discharge*	1.0(4)	1.0(2)
	*Skin disease (e.g. eczema)*	0.0(0)	1.0(2)
	*Irritability*	0.0(0)	0.5(1)

*P < 0.05 versus the Sub/MF59™ vaccine group; **P ≤ 0.005 versus the Subunit vaccine group

Sub/MF59™, MF59™-adjuvanted subunit influenza vaccine; Subunit, non-adjuvanted subunit influenza vaccine

### Immunogenicity

Pre-vaccination GMTs were similar for both vaccine groups against all three influenza strains tested. Baseline seroprotection rates were also similar between vaccine groups, with >97% of subjects in each vaccine cohort seroprotected against A/H1N1 (Sub/MF59™, 98.4%; Subunit, 97.3%), >18% against A/H3N2 (Sub/MF59™, 21.5%; Subunit, 18.7%) and >2% against B (Sub/MF59™, 2.2%; Subunit, 2.1%) prior to vaccination (Table 3).

**Table 3:** Pre- and post-vaccination seroprotection rate (percentage of subjects with haemagglutination inhibition titre ≥ 1:40)

**Viral strain**	**Vaccination status**	**Sub/MF59™, % (n = 367)**	**Subunit, % (n = 187)**
**A/H1N1**	Pre-vaccination	98.4	97.3
	Post-vaccination	99.7	99.5
**A/H3N2**	Pre-vaccination	21.5	18.7
	Post-vaccination	88.0*	72.2
**B**	Pre-vaccination	2.2	2.1
	Post-vaccination	35.7	28.3

*P < 0.001 versus the Subunit group

Sub/MF59™, MF59™-adjuvanted subunit influenza vaccine; Subunit, non-adjuvanted subunit influenza vaccine

**Figure 2:**
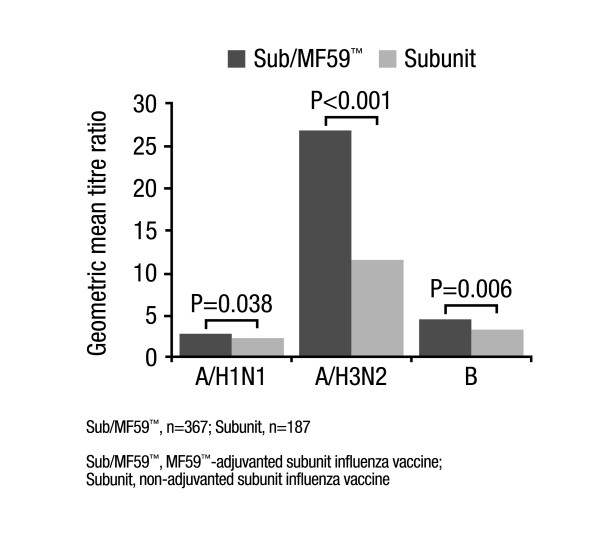
Geometric mean titre ratios (Day22:Day1) in all subjects in the Phase II/III trial.

**Figure 3:**
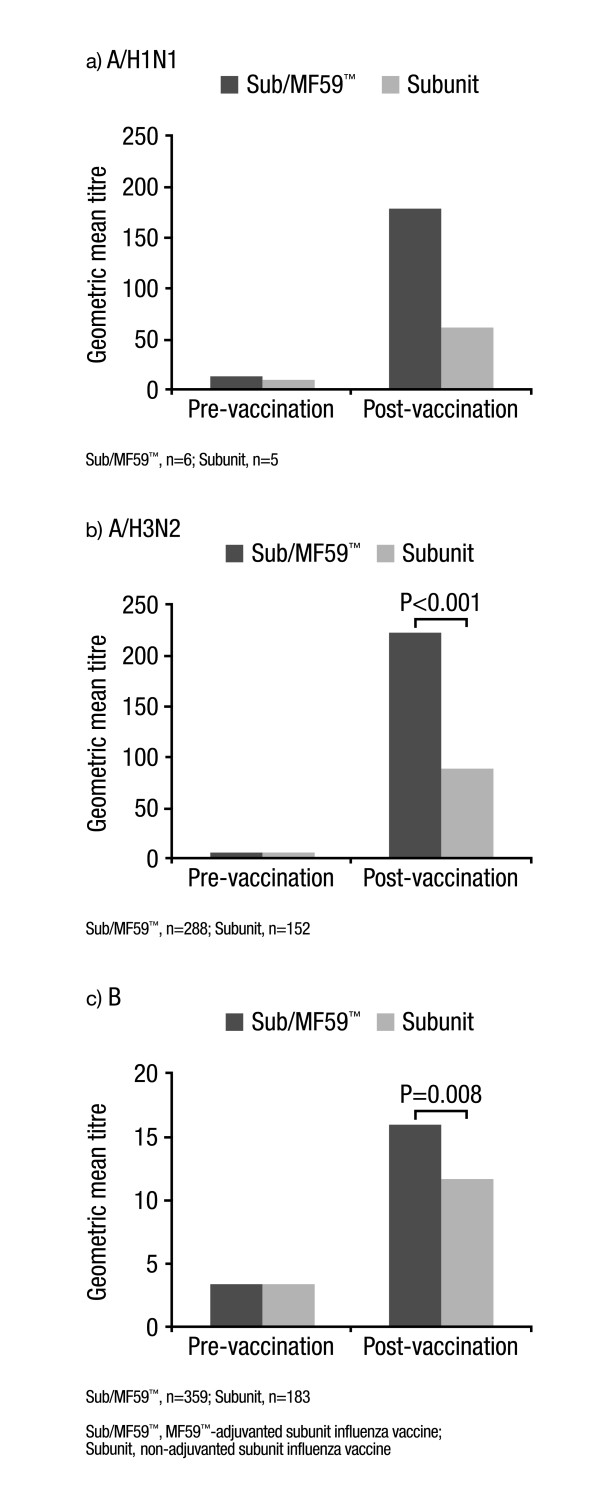
Pre-vaccination (Day 1) and post-vaccination (Day 22) geometric mean titre against a) A/H1N1, b) A/H3N2 and c) B viral strains in subjects without pre-vaccination immunoprotection.

At 3 weeks post-vaccination, significantly (P < 0.001) higher GMTs were reported for both vaccine groups versus baseline (data not shown); however, in the Sub/MF59™ group, post-vaccination GMTs against all three strains were significantly higher compared with the Subunit group (A/H1N1, 1439.01 versus 1197.39, respectively, P = 0.034; A/H3N2, 274.61 versus 110.85, respectively, P < 0.001; B, 16.59 versus 11.95, respectively, P = 0.005). Furthermore, GMT ratios (Day 22:Day 1) against the A/H1N1, A/H3N2 and B strains were significantly (P = 0.038, P < 0.001, P = 0.006, respectively) greater in the Sub/MF59™ group compared with the Subunit group (Figure 2). Following analysis of the data, including only those subjects who did not have seroprotective titres prior to vaccination, the same trend was found: higher post-vaccination GMTs were reported in the Sub/MF59™ group compared with the Subunit group for the A/H3N2 and B strains (P < 0.001 and P = 0.008, respectively), but not for A/H1N1 (Figure 3).

Post-vaccination, the percentage of subjects seroprotected against the A/H3N2 strain was significantly (P < 0.001) greater after vaccination with Sub/MF59™ compared with Subunit vaccination (Table 3). Although the seroprotection rate for all subjects against the A/H1N1 and B strains was higher in the Sub/MF59™ group, no significant difference was found between vaccine groups. In addition, a significantly (P < 0.001) greater number of previously unprotected elderly subjects in the Sub/MF59™ vaccine group demonstrated seroconversion against the A/H3N2 strain, compared with the Subunit group (85.1 versus 66.2%, respectively) (Table 4).

**Table 4:** Seroconversion rates (percentage of subjects with seroconversion^a ^or a significant increase in HI titres^b ^in subjects without pre-vaccination immunoprotection)

	**Seroconversion rates, %**	
**Viral strain**	**Sub/MF59™**	**Subunit**

**A/H1N1^c^**	83.3	80.0
**A/H3N2^d^**	85.1*	66.2
**B^e^**	33.4	25.8

^a^Seroconversion is defined as negative pre-vaccination serum (i.e. haemagglutinin inhibition [HI] titre <1:10) and post-vaccination HI titre ≥ 1:40; ^b^Significant increase is defined as ≥ 4-fold increase from non-negative (≥ 1:10) pre-vaccination HI titre; ^c^Number of subjects: Sub/MF59™, 6; Subunit, 5; ^d^Number of subjects: Sub/MF59™, 288; Subunit, 152; ^e^Number of subjects: Sub/MF59™, 359; Subunit, 183

*P < 0.001 versus the Subunit group

Sub/MF59™, MF59™-adjuvanted subunit influenza vaccine; Subunit, non-adjuvanted subunit influenza vaccine

## Discussion

This study has demonstrated that MF59™-adjuvanted subunit influenza vaccine is as well tolerated as non-adjuvanted subunit influenza vaccine in elderly Chinese subjects. Although the number and incidence of some of the solicited local reactions in the Sub/MF59™ vaccine group was greater than in the Subunit group, they were generally mild or moderate and of short duration, and no subjects withdrew from the study due to safety concerns. Furthermore, the incidence of injection-site pain reported here was lower than has been reported in other studies [25,27]. Although the incidence of fever was higher than has been reported previously with MF59™-adjuvanted influenza vaccination [25,27], the increased incidence was evident for both vaccines used in this study, rather than for the Sub/MF59™ vaccine only. The increase does not appear to be due to race, as in a previous study in elderly Chinese subjects [19] the incidence of fever was 0% following vaccination with non-adjuvanted influenza vaccine. These findings are in agreement with the published results of clinical trials conducted in elderly Caucasian populations in Europe [25]. These trials supported registration of the vaccine in Europe, and concluded that the addition of MF59™ to subunit influenza vaccines does not cause clinically important changes in the safety profile of the influenza vaccine. To date, more than 30 million doses of the MF59™-adjuvanted subunit influenza vaccine have been sold, and it has demonstrated a good safety profile [32]. Evaluation of post-marketing pharmacovigilance case reports (n = 385; September 1997 to April 2006) confirmed that vaccination with the MF59™-adjuvanted subunit influenza vaccine was associated with a very low frequency of adverse reactions [33].

Evaluation of the primary (pre- and post-vaccination GMTs and GMT ratio) and secondary (proportion of subjects with protective antibody titres [≥ 40] and those demonstrating seroconversion post-vaccination) immunogenicity parameters showed that both the Sub/MF59™ and Subunit vaccines were able to induce an immune response in elderly Chinese subjects. Significantly (P < 0.05) higher antibody titres were induced by the Sub/MF59™ vaccine compared with the Subunit vaccine. Furthermore, as for the safety profile, the immunogenicity results agree with the results of trials conducted in Europe [24,25,27]. For all three influenza strains tested, both the MF59™-adjuvanted and the non-adjuvanted vaccine met at least one criterion of the European Committee for Medicinal Products for Human Use (CHMP) criteria for the immunogenicity evaluation of seasonal influenza vaccines, as required for vaccine licensure [34]. For both vaccines, the trend was for higher immunogenicity for the influenza A strains, compared to the B strain. It is of note that very high pre-vaccination titres were recorded in this study, especially for A/H1N1. This could be explained in part by the A/H1N1 vaccine strain (A/New Caledonia/20/99-like H1N1) being included in the vaccine formulation for several influenza seasons previous to the 2005–2006 season. Furthermore, from an epidemiological standpoint, influenza A strains are often the most dominant and the most relevant strains among adults and the elderly, with A/H1N1 having been the predominant circulating strain in the Guangxi region of China during the time before the trial was conducted.

It is well documented that levels of HI antibody titres correlate with seroprotection against influenza [35], thus it is expected that increased immunogenicity should lead to increased clinical protection from influenza. This approach has been widely used across the clinical trial programme for the MF59™-adjuvanted vaccine [25]. During field studies in Europe, MF59™-adjuvanted vaccine has been shown to offer greater clinical protection against influenza-like illness compared with non-adjuvanted vaccine [26]. Therefore, it is anticipated that MF59™-adjuvanted vaccine may also offer greater clinical protection in elderly Chinese subjects.

Results from trials using an MF59™-adjuvanted subunit influenza vaccine suggest that adjuvanted vaccination induces a greater immune response in elderly (≥ 60 years of age) Chinese subjects, and demonstrates a good tolerability profile, compared with a non-adjuvanted subunit influenza vaccine.

## Conclusion

In conclusion, the study results strengthen support for the use of the MF59™-adjuvanted subunit influenza vaccine (FLUAD^®^) among the elderly population in China.

## Methods

### Study design

A preliminary Phase I, open-label study was conducted in February-March 2006 to assess the safety of an MF59™-adjuvanted subunit influenza vaccine (Sub/MF59™; FLUAD®, Novartis Vaccines) in elderly Chinese subjects. The study recruited 25 Chinese subjects (≥ 60 years of age); of these, 24 subjects were administered Sub/MF59™ in follow-up sequence.

Following this safety trial, a Phase II/III randomised, observer-blind, controlled study was conducted to assess the safety and immunogenicity of Sub/MF59™ in a large cohort of elderly (≥ 60 years of age) Chinese subjects. Subjects were randomised (2:1) to receive either Sub/MF59™ (n = 400) or a non-adjuvanted subunit influenza vaccine (Subunit; Agrippal®, Novartis Vaccines; n = 200). Both vaccines included the strains recommended for the 2005–2006 Northern hemisphere influenza season. Randomisation was achieved using the PEMS V2.1 statistical software (Statistics Teaching and Research Division, West China University of Medical Sciences; released January 1996). A 0.5 ml dose was administered intramuscularly in the deltoid region of the non-dominant arm.

### Study population

#### Inclusion criteria

Subjects invited to participate in the trial were those ≥ 60 years of age, who were healthy and willing/able to provide written informed consent prior to study entry.

#### Exclusion criteria

Subjects with underlying disease chronic, such as tumours, autoimmune diseases, progressive artherosclerosis or complicated diabetes mellitus, chronic obstructive pulmonary disorder requiring oxygen therapy, acute or progressive liver or renal disease, or congestive heart failure; subjects with a known allergy to any vaccine components; subjects with laboratory-confirmed influenza or vaccinated against influenza within 6 months prior to enrolment; subjects who had received any other vaccine or investigational agent within 4 weeks prior to enrolment; subjects with current infectious disease, including those taking systemic antibiotics or antivirals.

Both the pivotal safety study and the subsequent trial were performed according to the ethical guidelines of the 1975 Declaration of Helsinki, Good Clinical Practice, and local laws. Before the trials started, the study protocol and informed consent form were approved by local ethics committees; all subjects signed an Informed Consent Form.

### Objectives

#### Safety

Local and systemic reactions, including all adverse events (AEs), were monitored and recorded 14 days post-vaccination for the Phase I trial and 22 days post-vaccination for the Phase II/III trial, controlled study.

#### Immunogenicity

For the Phase II/III trial, blood samples were taken from all subjects pre-vaccination (Day 1) and post-vaccination (Day 22). Haemagglutinin inhibition (HI) antibody titres were measured in all samples. *Primary parameters*: Geometric mean titres (GMTs) against A/New Caledonia/20/99-like (A/H1N1), A/California/7/2004-like (A/H3N2) and B/Shanghai/361/2002-like influenza strains were measured for each vaccine pre- and post-vaccination (Day 1 and Day 22). The geometric mean titre ratio (GMR) of post-vaccination titres versus pre-vaccination titres was also reported. *Secondary parameters*: For each influenza strain, the number of subjects with seroprotective HI antibody titres (≥ 1:40) was also evaluated pre-vaccination and post-vaccination (Day 1 and Day 22), and the number of subjects achieving seroconversion (defined as ≥ 4-fold increase in HI titre from non-negative pre-vaccination titre [≥ 1:10] or a rise from <1:10 to a post-vaccination HI titre ≥ 1:40) post-vaccination (Day 22) was calculated.

### Statistical methods, analysis and objectives

Data were analysed using the SAS V8.2 software. Statistical significance between vaccine groups for data describing the population's baseline characteristics was calculated using the Student's t-test and the chi-square test, or Fisher's exact test where necessary. Analysis of covariance was performed to calculate the confidence intervals for GMTs at Day 22, with GMT at Day 1 as covariate. Paired t-tests were performed to assess the difference between pre- and post-vaccination GMTs within each vaccine group. A P-value of <0.05 was considered to indicate statistical significance.

## Competing interests

The study was funded by Novartis Vaccines, and Michele Pellegrini and Audino Podda are employees of Novartis Vaccines. The remaining authors declare that they have no competing interests.

## Authors' contributions

RL and HF were responsible for the design, co-ordination and conduct of the clinical trial and the statistical analysis. RL, HF, YL, YL, MP and AP consulted the clinical results and prepared the manuscript. All authors read and approved the final manuscript.
